# Identification of a conserved maxicircle and unique minicircles as part of the mitochondrial genome of *Leishmania martiniquensis* strain PCM3 in Thailand

**DOI:** 10.1186/s13071-022-05592-1

**Published:** 2022-12-12

**Authors:** Pornchai Anuntasomboon, Suradej Siripattanapipong, Sasimanas Unajak, Kiattawee Choowongkomon, Richard Burchmore, Saovanee Leelayoova, Mathirut Mungthin, Teerasak E-kobon

**Affiliations:** 1 Department of Genetics, Faculty of Science, Kasetsart University, Bangkok, Thailand; 2 Omics Center for Agriculture, Bioresources, Food, and Health, Kasetsart University (OmiKU), Bangkok, Thailand; 3 Department of Microbiology, Faculty of Science, Mahidol University, Bangkok, Thailand; 4 Department of Biochemistry, Faculty of Science, Kasetsart University, Bangkok, Thailand; 5 Glasgow Polyomics, College of Medical, Veterinary and Life Sciences, University of Glasgow, Glasgow, UK; 6 Department of Parasitology, Phramongkutklao College of Medicine, Bangkok, Thailand

**Keywords:** *Leishmania*, *Leishmania martiniquensis*, Kinetoplast, Maxicircle, Minicircle

## Abstract

**Background:**

The mitochondrial DNA of trypanosomatids, including *Leishmania*, is known as kinetoplast DNAs (kDNAs). The kDNAs form networks of hundreds of DNA circles that are evidently interlocked and require complex RNA editing. Previous studies showed that kDNA played a role in drug resistance, adaptation, and survival of *Leishmania*. *Leishmania martiniquensis* is one of the most frequently observed species in Thailand, and its kDNAs have not been illustrated.

**Methods:**

This study aimed to extract the kDNA sequences from Illumina short-read and PacBio long-read whole-genome sequence data of *L. martiniquensis* strain PCM3 priorly isolated from the southern province of Thailand. A circular maxicircle DNA was reconstructed by de novo assembly using the SPAdes program, while the minicircle sequences were retrieved and assembled by the rKOMIC tool. The kDNA contigs were confirmed by blasting to the NCBI database, followed by comparative genomic and phylogenetic analysis.

**Results:**

We successfully constructed the complete circular sequence of the maxicircle (19,008 bp) and 214 classes of the minicircles from *L. martiniquensis* strain PCM3. The genome comparison and annotation showed that the maxicircle structure of *L. martiniquensis* strain PCM3 was similar to those of *L. enriettii* strain LEM3045 (84.29%), *L. arabica* strain LEM1108 (82.79%), and *L. tarentolae* (79.2%). Phylogenetic analysis also showed unique evolution of the minicircles of *L. martiniquensis* strain PCM3 from other examined *Leishmania* species.

**Conclusions:**

This was the first report of the complete maxicircle and 214 minicircles of *L. martiniquensis* strain PCM3 using integrated whole-genome sequencing data. The information will be helpful for further improvement of diagnosis methods and monitoring genetic diversity changes of this parasite.

**Graphical abstract:**

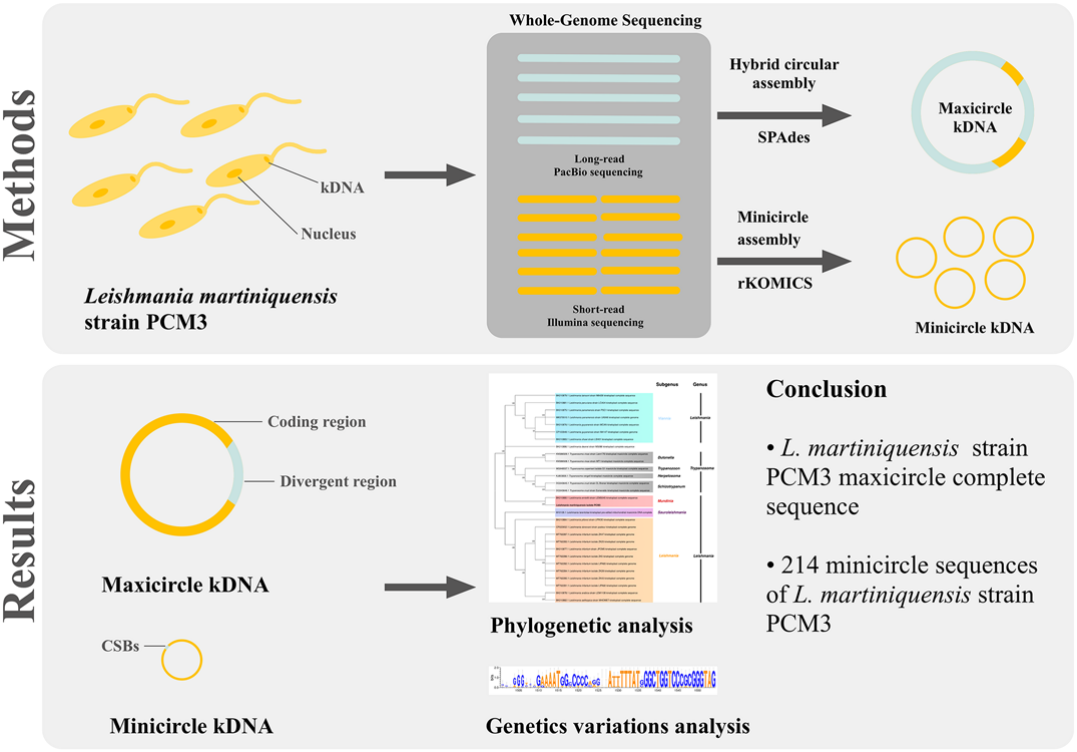

## Background

Leishmaniasis is a sandfly-borne disease in tropical and subtropical regions of the world caused by protozoan parasites of the genus *Leishmania*. At least 54 species have been identified to date, which could be divided into two lineages: (1) Euleishmania consisting of four subgenera, *Leishmania*, *Mundinia*, *Sauroleishmania*, and *Viannia*; (2) *Paraleishmania* [1]. In Thailand, leishmaniasis was considered to come from imported cases from patients who had visited the endemic areas since 1960; for example, an autochthonous visceral leishmaniasis case was detected in 1996 in a patient without underlying illness [2–4]. *Leishmania infantum* and *L. donovani* were frequently seen in these cases [3]. Until 2008, a new species, *Leishmania siamensis*, from a Thai patient in the southern province, was described and later renamed *Leishmania orientalis* [3–5]. In the same year, a suspected case was reported as visceral leishmaniasis in another patient in the southern province of Thailand. The case was confirmed to be *Leishmania martiniquensis*, a member of the *Leishmania enriettii* complex [3, 6, 7]. Since then, more cases of these two *Leishmania* species have been observed. In 2015, one case of *L. martiniquensis* and 12 cases of *L. orientalis* were detected in 392 participants from the northern province who showed no symptoms [8]. One *Phlebotomus stantoni* and one black rat also tested positive for *L. martiniquensis*. These have raised concerns about possible outbreaks of these *Leishmania* species and their impact on healthcare.

To better understand these parasites’ genetics, the chromosome-scale genomes of *L. orientalis* strain LSCM4 (34.19 Mbp) and *L. martiniquensis* strain LSCM1 (32.41 Mbp) isolated from patients in the northern province were published in 2021 [9–11]. Shortly after, in 2022, genomes of *L. orientalis* strain PCM2 (30.01 Mbp) and *L. martiniquensis* strains PCM3 (32.39 Mbp) and CU1 (30.80 Mbp) isolated from the southern provinces were released [12, 13]. Comparative genomic analysis of these *Leishmania* samples addressed the close relationship between the two *L. martiniquensis* strains, PCM3 and LSCM4, and further genetic distance between the two *L. orientalis* strains [12]. Similarly, a genomic comparison of two *L. martiniquesis* strains, CU1 and LSCM1, identified 50 genes unique to strain CU1 and ten genes to the strain LSCM1[13]. This nuclear genomic information has provided the basis for understanding the virulence and improving detection methods and drug development, e.g. identifying 16 species-specific protein therapeutic targets [14].

Kinetoplast DNA, or kDNA, is a substructure of complex DNA networks discovered within trypanosomatid mitochondria consisting of long maxicircles and minicircles [15–19]. The kDNAs of *L. tarentolae* have been widely used as a model for RNA editing studies [20–22]. A single mitochondrion of *L. tarentolae* is composed of 20–50 maxicircles (20–40 kb), which are equivalent to the mitochondrial DNA of other eukaryotes and 5,000–10,000 minicircles (0.5–2 kb) [23]. Maxicircle sequences were homogeneous within the parasite species [23]. The genes on the *L. tarentolae* maxicircle included four genes (*9S rRNA*, *12S rRNA*, *RPS12*, and *RPS3*) that encoded for rRNA and protein components of the mitochondrial ribosome and 16 genes (*ND1*, *ND3*, *ND4*, *ND5*, *ND*7, *ND8*, *ND9*, *COI*, CO*II*, *COIII*, *Cyb*, *ATPase6*, *MURF1*, *MURF2*, *CR3*, and *CR4*) involved in the electron transport chain complexes (GenBank; M10126.1). Many of the protein-coding maxicircle genes were transcribed as pre-mRNAs that must be modified post-transcriptionally via an extensive RNA editing process (i.e. insertion and deletion of uridine) to yield translatable mRNAs. The RNA editing process required several guide RNAs (gRNAs) encoded by classes of the minicircles with varying copy numbers to prevent the loss of particular types which could be lethal to the parasite [24]. The maxicircle also contained the divergent region (DR), which was non-coding and enriched with unique repeat patterns [20, 24–26]. Camacho et al. compared kDNAs of *L. major*, *L. infantum*, and *L. braziliensis* with the maxicircle of *L. tarentolae* and found that the maxicircles of these three species were highly conserved. At the same time, all minicircles shared the conserved CSB boxes, but the copy number differed (97 for *L. major*, 49 for *L. infantum*, and 3 for *L. braziliensis)* [27]. Ceccarelli et al. used the minicircle sequences to design PCR primers for distinguishing between *L. infatum* and *Leishmania amazonensis* [28]. Thus, the maxicircle and minicircle DNAs were useful for examining inter- and intra-specific relationships between *Leishmania* species and strains. However, the maxicircle and minicircle DNAs of *L. martiniquensis* strain PCM3 had not been identified. This study aimed to extract these kDNA sequences from the short- and long-read whole-genome sequence data and apply bioinformatics analysis to explore these genomes with other *Leishmania* species. Results filled the missing knowledge gaps of the *L. martiniquensis* genomics and helped understand the genetic diversity and virulence of the parasite.

## Materials and methods

### Culture of L. martiniquensis strain PCM3

*Leishmania martiniquensis* strain PCM3 was maintained and provided by the Department of Parasitology, Phramongkutklao College of Medicine, Bangkok, Thailand. The promastigotes were grown at 26 °C in RPMI 1640 modified with 13.3 mM glutamine, 2.5 mM arginine, 0.3 mM cysteine, 1.7 mM glutamate, 62.1 mM proline, 0.6 mM ornithine, 3.8 mM glucose, 2.2 mM fructose, 5.1 mM malate, 2.8 mM α-ketoglutarate, 0.5 mM fumarate, 0.5 mM succinate, 25 mM Hepes, 50 µg/ml gentamicin, 2X MEM vitamins (Gibco, US), and 20% heat-inactivated fetal bovine serum (HIFBS, Gibco, US).

### Genomic DNA preparation

Genomic DNA was isolated from the promastigotes at the late logarithmic phase. After washing with ultrapure water, the promastigote pellet was suspended in 1 ml of lysis buffer (10 mM Tris, 10 mM KCl, 10 mM MgCl_2_, 0.5 M NaCl, 2 mM EDTA, and 0.5% SDS) and 20 µl of proteinase K solution (20 mg/ml). After 30 min, the samples were incubated with chloroform: isoamyl alcohol (24:1) at 56 °C and gently mixed vigorously for 10 min. The samples were centrifuged at 14,000 × g for 10 min at room temperature to obtain the upper aqueous phase. Ten microliters of RNAse solution (20 mg/ml) was added and incubated at room temperature for 3 min. Following RNase treatment, a 1:1 ratio of chloroform:isoamyl alcohol (24:1, v/v) was added and gently mixed vigorously for 10 min before centrifugation at 14,000×*g* for 10 min at room temperature. The upper aqueous phase was collected, and DNAs were precipitated overnight at − 70 °C in 200 µl of 4 M ammonium acetate and 800 µl of 100% ethanol. The precipitated samples were centrifuged at 14,000×*g* for 10 min at 4 °C and then washed twice with 70% ethanol. After 30 min at room temperature, DNA samples were air-dried and suspended in TE buffer [10 mM Tris–HCl (pH 8.0) and 0.1 mM EDTA]. The extracted genomic DNA for the long-read sequencing was further purified using the phenol/chloroform method. The quality and amount of DNA were determined by measuring absorbance at 260/280 nm and 260 nm using Nanodrop (Thermo Fisher Scientific, US). Electrophoresis on a 1% agarose gel was used to determine the genomic integrity. Before genome sequencing, the samples were maintained at − 70 °C.

### Leishmania maxicircle and minicircle sequence extraction from raw sequence reads

For the short-read sequencing, a 101-bp pair-end read library was constructed for the whole genome sequencing using the Illumina HiSeq2000 platform (Illumina, USA). For the long-read sequencing, the 20-kb PacBio library was built according to the PacBio standard library preparation methods (Pacific Biosciences, CA, USA) using the SMRTbell Express Template Preparation kit 1.0. It was sequenced by the SMRTbell platform (Pacific Biosciences, USA). The Illumina reads were filtered using the BBTools program (http://jgi.doe.gov/data-and-tools/bb-tools) with the following parameter setting—ktrim = r [right-trimming (3′ adapters)] k = 23 mink = 11 (allows shorter kmers at the ends of the read) hdist = 1 (hamming distance) tpe (trim both reads) tbo qtrim = rl (trim the left and right sides) trimq = 10 (quality-trim to Q10) phiX (Illumina spikein)—to remove Illumina artifacts. The phiX sequences from bbtools library were used with 31-mer match (k = 31). To remove Illumina adapters, the UniVec database (https://www.ncbi.nlm.nih.gov/tools/vecscreen/univec/) was used as a reference for the adaptor sequences. These reads were used to generate hybrid assemblies for circular DNA by the SPAdes assembler version 3.15.3 [29, 30]; parameters were set as –pacbio–plasmid (assembly only circular DNA). Contigs were analyzed by BLASTn searches on the NCBI database. The BLAST results showed E-values < 0.01, and percentage of coverage > 75% was selected. The contigs sharing sequence homology with the complete maxicircle sequence of *Leishmania* was chosen for further analyses. For reference preparations, in-house python script was used to collect complete sequence of maxicircles, minicircles, and specific regions of *Leishmania* and *Trypanosoma* from the NCBI database. For the minicircles, the rKOMICS package was used for minicircle polishing, extending, and circularization [31], BLASTn scores were used to calculate the average nucleotide identity (ANI) using the pyani package [32], and percentage of similarity was represented by heatmap using the heatmaply package [33].

### Annotation and visualization of maxicircle and minicircle sequences

The BLASTn alignment was used to find the most similar *Leishmania* maxicircle sequence in the NCBI nucleotide database [34–42]. The extracted maxicircle contigs were annotated using manual annotation. The gene order of the maxicircle DNAs (*12S rRNA*, *9S rRNA*, *ND8*, *ND9*, *MURF5*, *ND7*, *CO3*, *Cyb*, *ATPase 6*, *ND2*, *G3*, *ND1*, *CO2*, *MURF2*, *CO1*, *G4*, *ND4*, *G5* or *ND3*, *RPS12*, and *ND5*) of *Leishmania arabica* strain LEM1108 (GenBank: BK010878) and *L. enriettii* strain LEM3045 (GenBank: BK010880) were used as references to construct the complete circular maxicircle genome of *L. martiniquensis* strain PCM3. Pairwise comparison of the reference and *L. martiniquensis* maxicircle sequences was conducted by the EMBOSS Water program (https://www.ebi.ac.uk/Tools/psa/emboss_water/) using the following parameters—EDNAFULL for matrix, gap penalty of 10.0, and extend penalty of 0.5—to identify gene location. Visualization and exploration of the maxicircle genome were done by the CGview program on the Proksee server [43] and the progressiveMauve alignment program [44]. Sequence read coverage of the maxicircle of *L. martiniquensis* strain PCM3 was analysed by using the samtools program version 1.15.1 [45, 46] and the SeqMonk program version 1.48.1 (https://www.bioinformatics.babraham.ac.uk/projects/seqmonk/). For the minicircle, the *L. martiniquensis* minicircle sequences were compared against the NCBI database for identification of closely related minicircles in other *Leishmania* species. Results with the E-value < 0.001 and percentage of coverage > 20% were collected and illustrated by the Sankey diagram using the SankeyMATIC program [47].

### Phylogenetic analysis of the obtained maxicircles and minicircles

Phylogenetic trees were built from the coding region of the maxicircle DNAs of available *Leishmania* and *Trypanosoma* species in the NCBI database compared with that of *L. martiniquensis*, similar to the method described by Solana et al. [48]. For minicircles, only universal minicircle invariant (CSB-3, CSB-1 and CSB-2) regions from *L. martiniquensis* strain PCM3 were compared with those available from the NCBI database. MAFFT program version 7 [49] on the Unipro UGENE version 44.0 [50] was used to align the sequences using the DNA gap open penalty of 1.53 and the DNA gap extension penalty of 0.123, and the manual refinements were omitted. Phylogenetic relationships were inferred by using the randomized axelerated maximum likelihood methods with bootstrap values of 10,000 replicates using the RAxML tool version 8 [51] and the approximately-maximum-likelihood method from the FastTree program version 2.1 [52] with local-bootstrap support. The labels on the phylogenetic tree were modified using the MEGA 11 program [53].

### Conserved motif analysis of the L. martiniquensis minicircles

The minicircle contigs from *L. martiniquensis* strain PCM3 were compared by multiple sequence alignment using the MAFFT program**.** The conserved motifs were annotated using data from previous studies and visualized by the WebLOGO3 program version 3.7 [54–56].

## Results

**Fig. 1:**
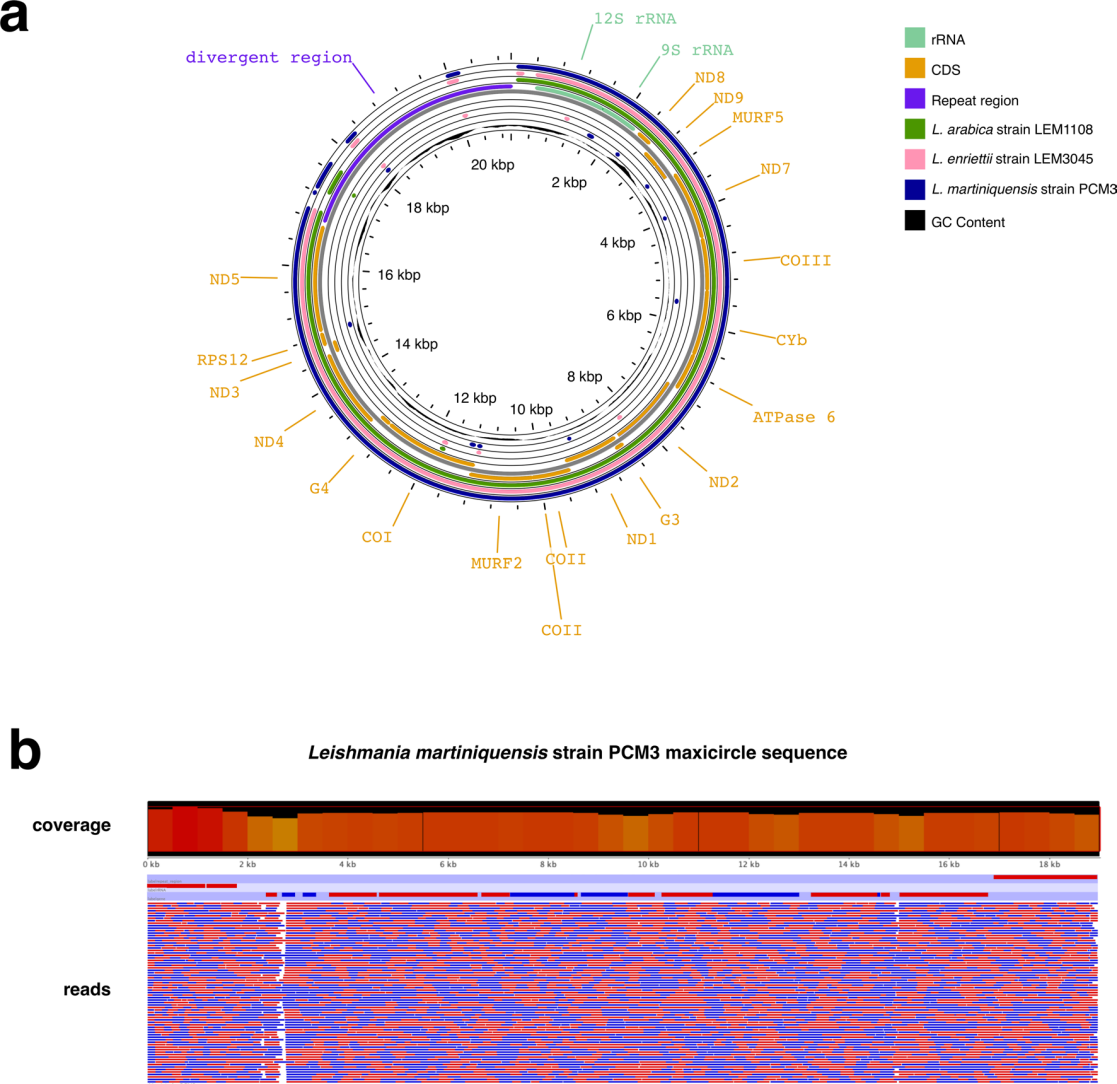
Circular diagram (**a**) represented comparison of the maxicircle DNAs of *L. martiniquensis* strain PCM3 (blue circle) and *L. enriettii* strain LEM3045 (pink circle) compared to the reference maxicircle genome of *L. arabica* strain LEM1108 (green circle). The reference annotation is shown in the outermost labels and the inner rings before the GC content. The genome annotation included coding sequence (CDS) region (orange), ribosomal RNA (rRNA) (light green), divergent region (purple), and GC content (black). The mapping of long-read data to the assembled maxicircle DNA of *L. martiniquensis* (**b**) showed a high level of sequence coverage (bar chart) and read depth (blue and red rectangles indicated different read directions)

This study successfully extracted the complete mitochondrial genome (maxicircle and minicircles) of *L. martiniquensis* strain PCM3 isolated from the southern province of Thailand by utilising both short- and long-read sequence data. The de novo assembly of the maxicircle genome resulted in a complete circular DNA scaffold with a length of 19,008 base pairs. The *L. martiniquensis* maxicircle contigs were highly similar to the complete maxicircle sequences of *Leishmania* species within the same subgenus *Mundinia* including *L. enriettii* strain LEM3045 (GenBank: BK010880.1), which had a length of 17,999 base pairs with the e-value of 0.0, 86% sequence coverage, and 84.29% identity, and another subgenus including *L. arabica* strain LEM1108 (GenBank: BK010878.1), which had a length of 17,536 base pairs at the e-value of 0.0, 87% sequence coverage, and 82.17% identity, and *L. tarentolae* (GenBank: M10126.1), which had a length of 20,992 base pairs at the e-value of 0.0, 86% sequence coverage, and 83.32% identity (Fig. 1a). The pairwise alignment of the maxicircle sequences of *L. martiniquensis* strain PCM3 with the reference sequences of *L. arabica* strain LEM1108, *L. enriettii* strain LEM3045, and *L. tarentolae* gave similar percentage of identity (79.2, 80.6, and 79.2%) and similarity (79.92, 80.6, and 79.2%) with 8.5, 7.0, and 7.2% gaps. The same rRNA and coding genes (CDS) were well conserved in the maxicircles of these three *Leishmania* species, whereas the divergent region (DR or repeat region) varied in sequence composition and length with a few fragments shared between the three species. The completeness of the maxicircle DNA of *L. martiniquensis* strain PCM3 was clearly showed by the high level of sequence coverage and read depth after re-mapping of the long-read data back to the assembled contigs (Fig. 1b). The sequence read coverage of 100% also verified validity of the maxicircle of *L. martiniquensis* strain PCM3 (Fig. 1b).

**Fig. 2:**
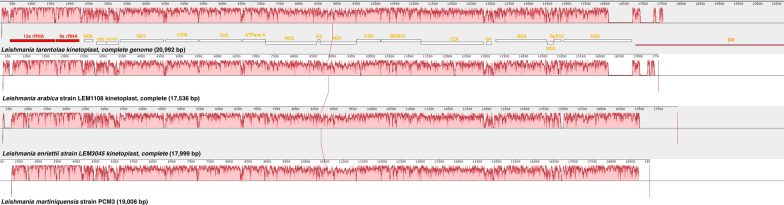
Comparative gene arrangement of maxicircles from four *Leishmania* species including *L. tarentolae* (first), *L. arabica* strain LEM1108 (second), *L. enriettii* strain LEM3045 (third), and *L. martiniquensis* strain PCM3 (last) using the progressive Mauve alignment program. Arrangement of the maxicircle genes is shown in red boxes for rRNA, white boxes for coding gene, and light red boxes for divergent region. The percentage of sequence similarity is represented in the red histograms

The gene arrangement on the maxicircle of *L. martiniquensis* strain PCM3 was compared with those of *L. enriettii* strain LEM3045, *L. arabica* strain LEM1108, and *L. tarentolae* (Fig. 2). The four *Leishmania* species shared conserved gene arrangement of 20 coding genes ordered as *12S rRNA*, *9S rRNA*, *ND8*, *ND9*, *MURF5*, *ND7*, *COIII*, *Cyb*, *ATPase 6*, *ND2*, *G3*, *ND1*, *COII*, *MURF2*, *COI*, *G4*, *ND4*, *ND3*, *RpS12*, and *ND5*. The maxicircle variation could be observed across the four *Leishmania* species, particularly in the intergenic and DR regions.

**Fig. 3:**
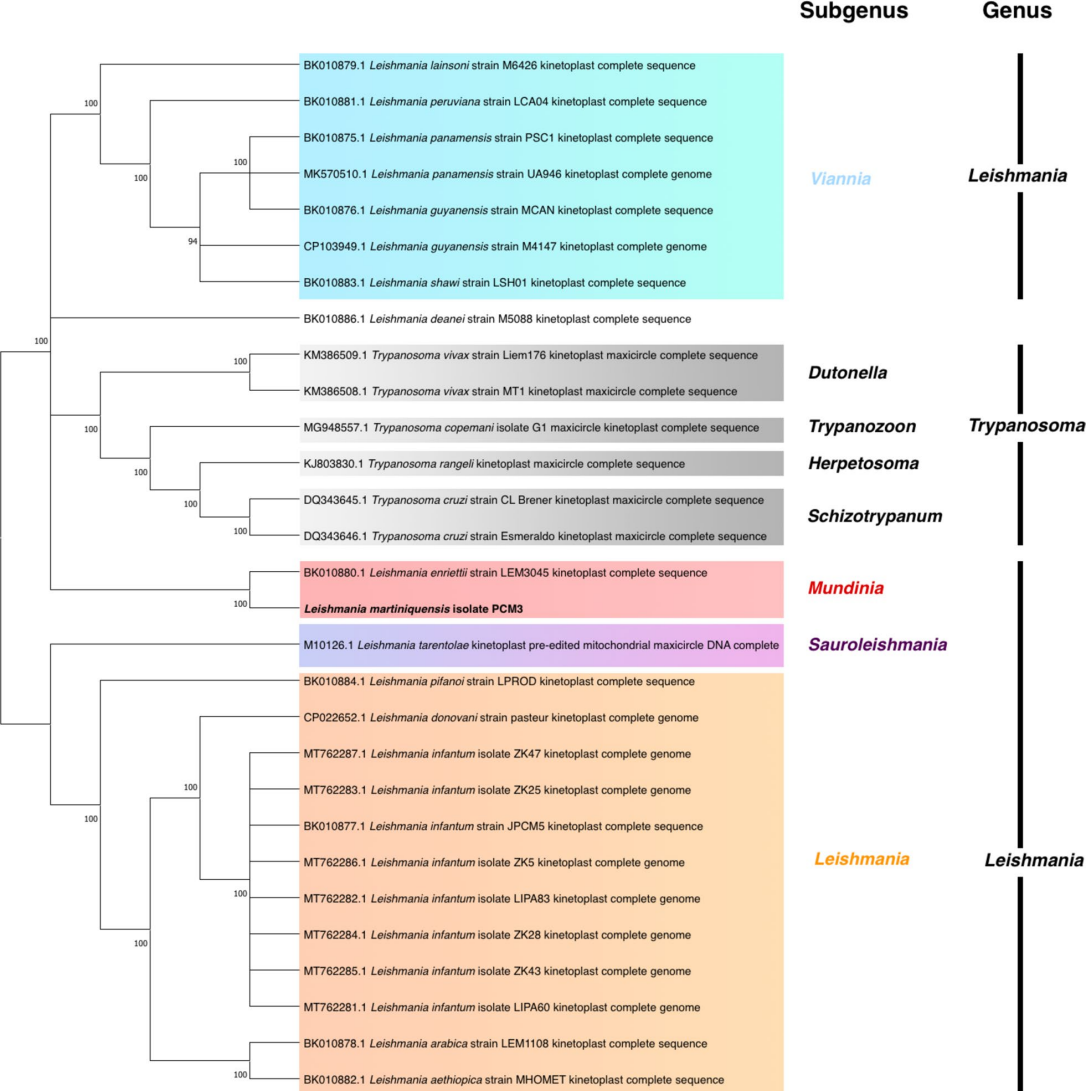
Phylogenetic tree based on the coding regions of the maxicircle DNA of *L. martiniquensis* strain PCM3 compared with 22 maxicircles of 13 *Leishmania* species and 6 maxicircles of *Trypanosoma vivax, Trypanosoma copemani*, *Trypanosoma rangeli*, and *Trypanosoma cruzi* from the NCBI nucleotide database. The randomized axelerated maximum likelihood method with the GTR + G4 model was used in the phylogenetic analysis. The branch length was the proportion of trees in which the linked taxa were grouped, and the bootstrap values were derived from 10,000 repetitions. Bootstrap values < 90 were not presented

Coding region-based phylogenetic analysis of the maxicircle of *L. martiniquensis* strain PCM3 compared with 28 complete maxicircles (22 *Leishmania* and 6 *Trypanosoma* samples) showed that this region could be used to categorize *Leishmania* samples into four subgenera: *Viennia*, *Mundinia*, *Sauroleishmania*, and *Leishmania*, separately from the genus *Trypanosoma* including subgenera *Dutonella*, *Trypanozoon*, *Herpetosoma*, and *Schizotrypanum* (Fig. 3). The maxicircle of *L. matiniquensis* strain PCM3 was correctly grouped with that of *L. enriettii* in the same subgenus *Mundinia*. This study also explored the phylogenetic relationship using the highly variable DR region, and the result was inconclusive.

**Fig. 4:**
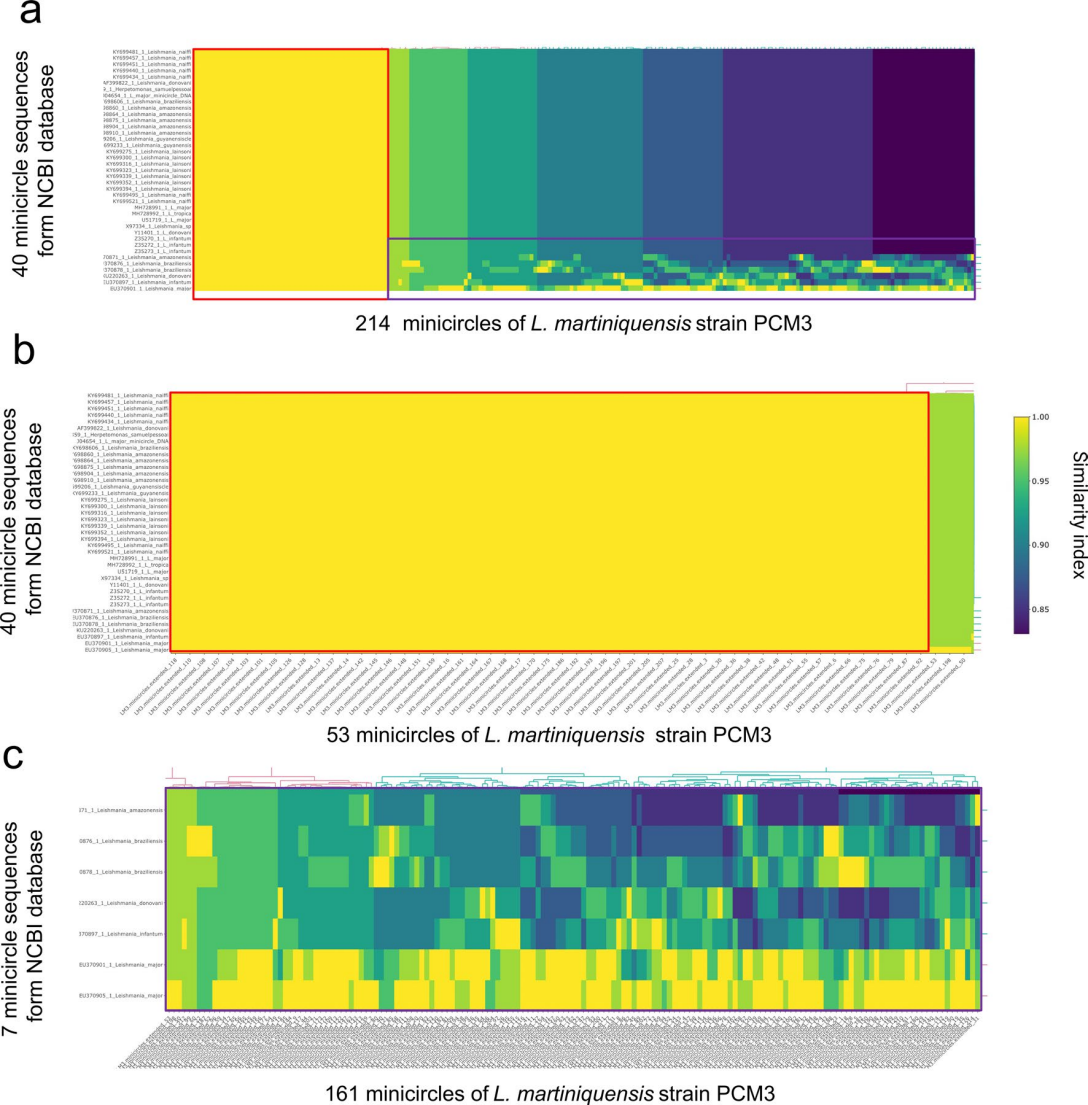
Heatmap representing the average nucleotide identity blast (ANIb) values of the 214 minicircle sequences of *L. martiniquensis* strain PCM3 compared with 40 minicircle sequences of other *Leishmania* species and *Herpetomonas samuelpessoai* (**a**). The ANIb value was obtained by the pyani tools in the blastn alignment mode. The heatmap was created using the Heatmaply program and the basic ANIb identity value received from the pyani package output. The red box area in (**a**) was enlarged in (**b**) to show detail of the 53 minicircle sequences of *L. martiniquensis* strain PCM3 that were highly matched to all 40 *Leishmania* minicircles from the database. The purple box area in (**a**) was expanded in (**c**) to show the remaining minicircles that were variably compared to those of other *Leishmania* species

**Fig. 5:**
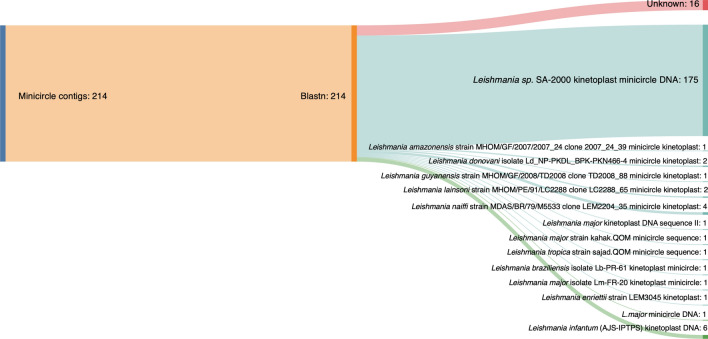
Sankey diagram summarized the blastn comparison of 214 minicircle contigs of *L. martiniquensis* strain PCM3 with other minicircles in the NCBI nucleotide database. The orange-colored strip represents these 214 minicircles that were closely matched to the minicircles of different *Leishmania* species including the novel minicircles (i.e. pink, blue, and green stripes)

For the minicircles, this study identified 214 minicircles (Fig. 4a) from *L. martiniquensis* strain PCM3 with length from 400 to 3385 base pairs (an average length of 717 base pairs). These were divided into 32 complete circular and 182 partial minicircles. The comparison of the average nucleotide identity (ANI) values showed that 53 minicircles were highly conserved in the examined *Leishmania* species (Fig. 4a, b), and 161 minicircles differed considerably from 40 minicircle sequences of 12 other *Leishmania* species and *Herpetomonas samuelpessoai* from the NCBI nucleotide database (Fig. 4a, c). The blastn comparison of these 214 minicircles showed that 175 contigs were similar to *Leishmania* sp. SA-2000 (GenBank: AF275904.1), which was the unclassified *Leishmania* species, six contigs were matched to *L. infantum* (AJS-IPTPS) minicircles (GenBank: Z35273.1), and four contigs were similar to those of *L. naiffi* (Fig. 5). A few contigs were similar to eight other *Leishmania* species and strains including *L. amazonensis* (1), *L. donovani* (2), *L. guyanensis* (1), *L. lainsoni* (2), *L. major* (4), *L. tropica* (1), *L. braziliensis* (1), and *L. enriettii* (1). Interestingly, 16 minicircles were unknown and could be specific to *L. martiniquensis*.

**Fig. 6:**
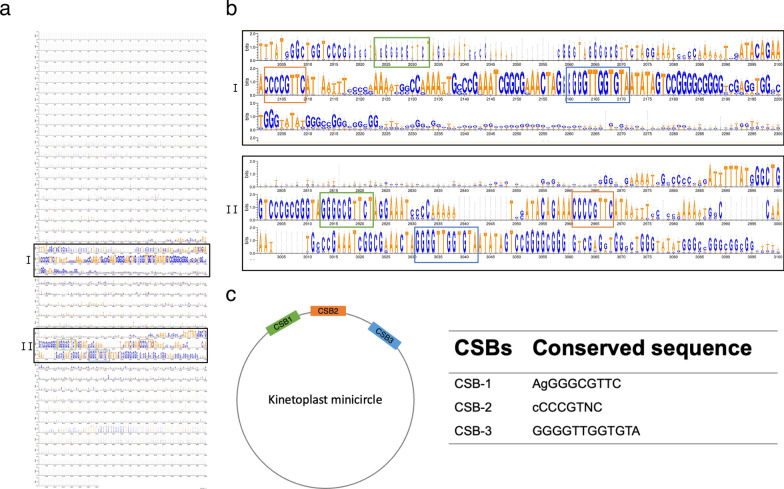
Conserved sequence box (CSB) analysis of the minicircles assembled from *L. martiniquensis* strain PCM3. (**a**) Multiple sequence alignments of 214 minicircle contigs generated by using ClustalOmega and visualized by the WebLogo 3 server. Two conserved regions were labeled as I and II. The height of the nucleotide character represented the level of conservation. (**b**) The conserved motifs within the two conserved regions of the *Leishmania* minicircles are highlighted by a green box (CSB1), an orange box (CSB2), and a blue box (CSB3). (**c**) The CSBs are shown in the diagrammatic representation along with their conserved sequences in the table

**Fig. 7:**
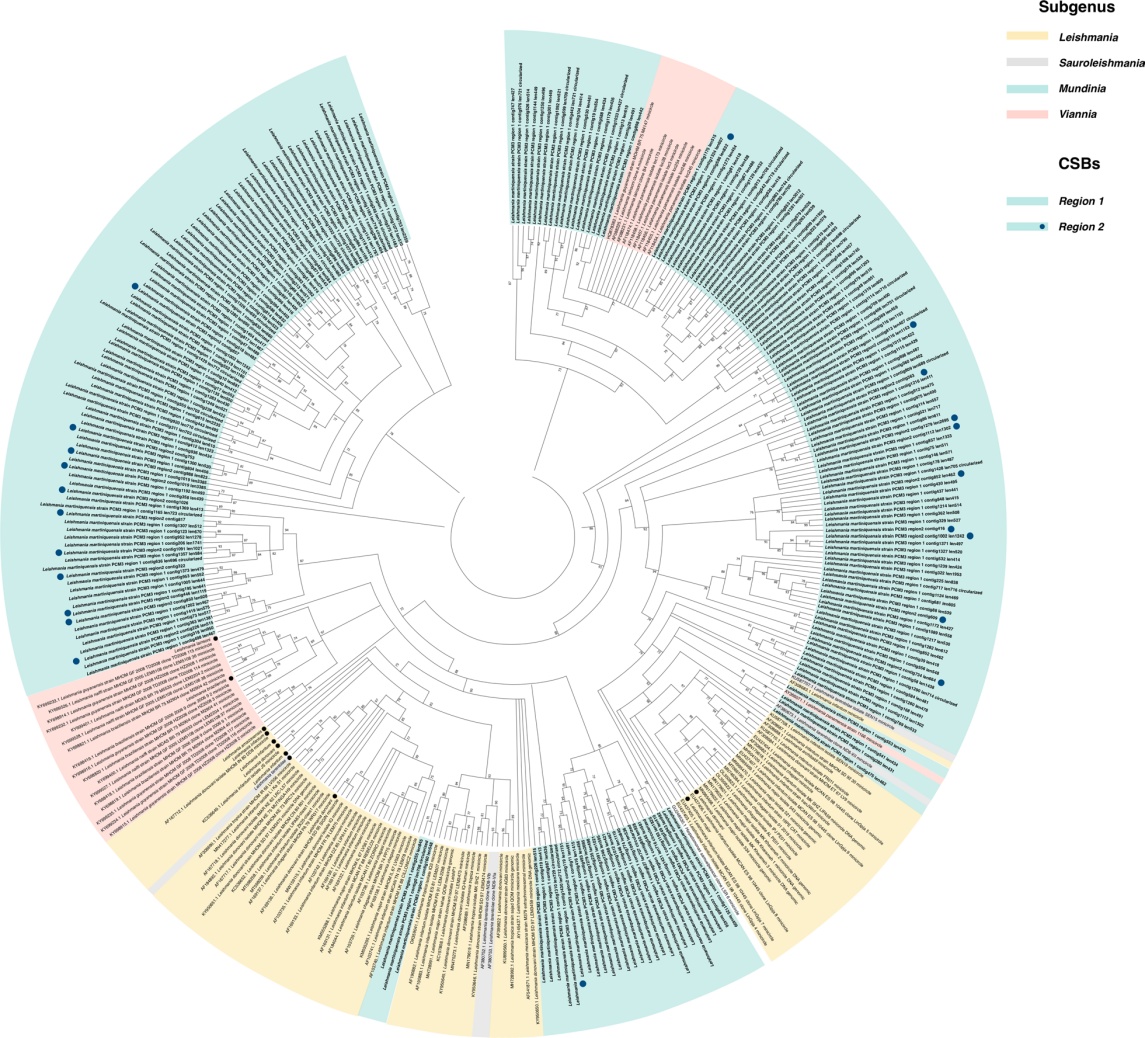
Phylogenetic tree of universal minicircle invariant regions (CSB-1, CSB-2, and CSB-3) from 371 minicircles was constructed by using approximate maximum-likelihood method of the FastTree program. 156 *Leishmania* minicircle sequences and one minicircle sequence of *Herpetomonas samuelpessoai* as (an outgroup) were collected from the NCBI database and compared with 214 minicircles of *L. martiniquensis* strain PCM3. Branches of the same species were collapsed. The local-bootstrap support values are shown at the branch point. Numbers < 70 are not presented. The tips are highlighted according to the genera: *Mundinia* (blue), *Leishmania* (orange), *Sauroleishmania* (purple), and *Viannia* (pink). In *Mundinia*, the tips without blue dots are the from conserved region I, and the tips with blue dots are the conserved region II. The black dot at the beginning of the tip name represents the collapsed clade members

Sequence comparison of 214 *L. martiniquensis* minicircles showed two major conserved regions (Fig. 6a) and the hypervariable regions. The conserved regions I and II contained three highly conserved sequence boxes, which were characteristic of all examined minicircles (Fig. 6b). These CSBs included 10-bp CSB-1 (AgGGGCGTTC), 8-bp CSB-2 (cCCCGTNC), and 12-bp CSB-3 (GGGGTTGGTGTA) (Fig. 6c). The presence of these motifs and their order confirmed the correct minicircle identification. Phylogenetic comparison was made of the conserved regions of 214 minicircle sequences of *L. martiniquensis* strain PCM3 with 156 *Leishmania* and one *Herpetomonas samuelpessoai* minicircles from the NCBI database. The constructed tree revealed certain degrees of minicircle conservation compared with other *Leishmania* species (Fig. 7). Most *L. martiniquensis* minicircles were grouped separately into their own clades (five main clades highlighted as blue tips of the tree in Fig. 7), and some were within the minor clades of *L. tropica*, *L. tarentolae*, *L. panamensis*, *L. lainsoni*, *L. guyanensis*, and *L. infantum*, similar to the result showed in Figs. 4 and 5. As 22 minicircles of *L. martiniquensis* strain PCM3 had conserved CSB regions I and II, phylogenetic analysis of these regions showed that all of them were dissimilar and distantly separated (Fig. 7).

## Discussion

The mitochondrial genome or kinetoplastid DNA (one maxicircle and 214 minicircles) of *L. martiniquensis* strain PCM3 was obtained from the hybrid assembly of the short- and long-read sequence data. Due to the length of *Leishmania* maxicircles between 20 and 40 kilobases [23], the use of both long- and short-read sequence data facilitated better reconstruction of the complete maxicircle and multiple minicircle DNA (including some complete minicircles). The DR region contains repetitive sequences and is known to be difficult during the assembly process [20, 57, 58]. The use of long-read data provided larger coverage of the entire DR region, thus resolving the complexity issue in assembling this region and assisting the maxicircle completion (Fig. 1b).

This finding provided a complete overview of this parasite genomics and complemented previously published nuclear genomic data of *L. martiniquensis* strain PCM3 [12]. The complete and partial maxicircle DNA (i.e. selected genes, all coding regions) could be used as genetic markers to identify *Leishmania* species and strains and to depict phylogenetic relatedness [59–62]. For example, Kaufer et al. used a variation on the *Leishmania* maxicircle sequences (*ND7* gene) to design PCR–RFLP assays to discriminate nine *Leishmania* species [63]. A recent study identified the origin of the intra-species *L. donovani* hybrid from Himachal Pradesh, India, that was derived from two independent *L. donovani* parents of the ISC1-Yeti clade from the Nepalese highlands using maxicircle phylogenetics, suggesting the usefulness for examination of intra-species diversity [64]. Similarly, this study showed that the *L. martiniquensis* maxicircle was highly homologous to *L. enriettii* and *L. arabica* (Fig. 1), and the gene arrangement of the *L. martiniquensis* maxicircle supported the genetic relationship with *L. enriettii*, suggesting the use of maxicircles to explore the genetic diversity of these *Leishmania* species (Fig. 2) [65]. This was also clearly supported by the close phylogenetic relationship of the maxicircle of *L. martiniquensis* strain PCM3 and *L. enriettii* strain LEM3045 from the same subgenus *Mundinia* (Fig. 3), consistent with the work of Solana et al., which also used the coding regions of maxicircle DNA [48]; however, the maxicircle sequences of subgenus *Mundinia* in the database remained limited and required further data gathering. Although the divergent region of *L. martiniquensis* maxicircle might not be suitable for the phylogenetic inference, the distinctiveness of this region was helpful in identification of *Leishmania* species and exploring intra-species variation. Therefore, the maxicircle data would benefit from further screening of *L. martiniquensis* isolates and other *Mundinia* members in areas of concern such as the Southeast Asian region where *L. martiniquensis* cases have been detected and observed more frequently than in the past (unpublished data).

*Leishmania martiniquensis* strain PCM3 had 214 minicircles which were considerably higher than those of *L. major* (97 classes), *L. infantum* (49 classes), and *L. braziliensis* (3 classes) and were lower than those of *Trypanosoma cruzi* (286 classes) [66]. These minicircles shared three conserved sequence blocks (CSB1-3), which acted as replication origins to encode short guide RNAs essential for editing encoded transcripts from their maxicircle [56, 67]. The similarity of most *L. martiniquensis* minicircles to the unclassified *Leishmania* species (*Leishmania* sp. SA-2000) would suggest that their phenotypic and genetic relationship should be further examined (Figs. 4, 5) and the proposed possibility that *Leishmania* sp. SA-2000 would be another strain of *L. martiniquensis*. Novel 16 minicircles were unique and could be specific to *L. martiniquensis* or particularly the PCM3 strain. These unique minicircles could be associated with virulence and drug resistance, e.g. the resistant *L. infantum* strain ZK47 had a specific minicircle pattern and increased copy number [68]. Furthermore, the presence of conserved regions I and II (Fig. 6), which contained two sets of the CSB-1, CSB-2, and CSB-3 conserved sequences in *L. martiniquensis* strain PCM3, was consistent with the previous observation of the conserved domain number variation in Trypanosomatid minicircles, which may be associated with the maturation of the maxicircle-encoded genes and the parasite adaptation [66, 69]. The phylogenetic analysis of the *L. matiniquensis* minicircles showed that most minicircles were classified separately into different clades and clustered distinctively from those of other *Leishmania* species, suggesting their paraphyletic origins. The results suggested potential usage of minicircle DNA sequence and number in identification of the *L. martiniquensis* species. For instance, the minicircles were also used to investigate the origin of the hybrid *L. braziliensis* × *Leishmania peruviana* isolates and found that their minicircles were derived from both parental species, while the maxicircle was uniparental [70]. Another study designed PCR primers specific to the conserved region of minicircle DNA coupled with high-throughput sequencing and identified different minicircle numbers, i.e. 62 for *L. amazonensis*, 133 for *L. braziliensis*, 196 for *L. guyanensis*, 94 for *L. infantum*, 97 for *L. lainsoni* and 88 for *L. naiffi*, which were used for inter- and intra-species genetic distance calculation [71]. Their minicircle patterns were applied to examine unknown *Leishmani* samples, and they correctly identified 10 out of 18 samples as *L. amazonensis*, *L. infantum*, *L. lainsoni*, and *L. naiffi*. Hence, not only the *L. martiniquensis* maxicircle, but also the sequence and number of minicircles would assist further diagnostics and screening of this parasite variant and population similar to previous studies in human patients and animals [72, 73].

The kDNAs of *L. martiniquensis* strain PCM3 in this study could provide the basis for understanding drug resistance phenotype. The alteration of kDNA by non-DNA-binding drugs such as sodium arsenite, tunicamycin, and pentamidine could lead to a phenomenon known as “transkinetoplastidy,” referring to dramatic changes in the population of maxicircles and minicircles [74, 75]. The kinetoplast study in *Trypanosoma congolense* revealed that mitochondrial membrane potential appeared to be involved in phenanthridine uptake, presumably via driving the accumulation of cationic compounds. When drug selection pressure was applied, populations with a lower mitochondrial electrical potential had an advantage because of isometamidium accumulation and lower toxicity [76]. It has been demonstrated that trypanocidal chemotherapies and their related chemicals also accumulated in the mitochondrion and interacted with the kDNAs [77]. Previous research also suggested that the *Leishmania* kinetoplast network may play a role in *Leishmania* and *Trypanosoma* survival [78–81]. Therefore, the kDNAs of *L. martiniquensis* strain PCM3 will benefit further experimental design to examine the association with drug-resistant and virulent phenotypes.

## Conclusion

This study reported the maxicircle and minicircle sequences of *L. martiniquensis* strain PCM3 isolated from the southern province of Thailand. Analysis of the short- and long-read sequence data revealed conserved structure and gene arrangement of the maxicircle, while 214 minicircles were mostly similar to the unclassified *Leishmania* species. Variation of these minicircles could be essential to the parasite’s survival and adaptation. These kDNAs have completed the gaps in understanding of the *L. martiniquensis* genetics and are helpful for inter- and intra-species identification.

## Data Availability

The data supporting this study’s findings are submitted to the NCBI GenBank database and will be openly available in the NCBI GenBank database, the BioProject ID: PRJNA863057 and the locus tag prefix: NP941.
